# Laser-Modified Surface Enhances Osseointegration and Biomechanical Anchorage of Commercially Pure Titanium Implants for Bone-Anchored Hearing Systems

**DOI:** 10.1371/journal.pone.0157504

**Published:** 2016-06-14

**Authors:** Furqan A. Shah, Martin L. Johansson, Omar Omar, Hanna Simonsson, Anders Palmquist, Peter Thomsen

**Affiliations:** 1 Department of Biomaterials, Institute of Clinical Sciences, Sahlgrenska Academy at University of Gothenburg, Göteborg, Sweden; 2 BIOMATCELL VINN Excellence Center of Biomaterials and Cell Therapy, Gothenburg, Sweden; 3 Oticon Medical AB, Askim, Sweden; University of Akron, UNITED STATES

## Abstract

Osseointegrated implants inserted in the temporal bone are a vital component of bone-anchored hearing systems (BAHS). Despite low implant failure levels, early loading protocols and simplified procedures necessitate the application of implants which promote bone formation, bone bonding and biomechanical stability. Here, screw-shaped, commercially pure titanium implants were selectively laser ablated within the thread valley using an Nd:YAG laser to produce a microtopography with a superimposed nanotexture and a thickened surface oxide layer. State-of-the-art machined implants served as controls. After eight weeks’ implantation in rabbit tibiae, resonance frequency analysis (RFA) values increased from insertion to retrieval for both implant types, while removal torque (RTQ) measurements showed 153% higher biomechanical anchorage of the laser-modified implants. Comparably high bone area (BA) and bone-implant contact (BIC) were recorded for both implant types but with distinctly different failure patterns following biomechanical testing. Fracture lines appeared within the bone ~30–50 μm from the laser-modified surface, while separation occurred at the bone-implant interface for the machined surface. Strong correlations were found between RTQ and BIC and between RFA at retrieval and BA. In the endosteal threads, where all the bone had formed *de novo*, the extracellular matrix composition, the mineralised bone area and osteocyte densities were comparable for the two types of implant. Using resin cast etching, osteocyte canaliculi were observed directly approaching the laser-modified implant surface. Transmission electron microscopy showed canaliculi in close proximity to the laser-modified surface, in addition to a highly ordered arrangement of collagen fibrils aligned parallel to the implant surface contour. It is concluded that the physico-chemical surface properties of laser-modified surfaces (thicker oxide, micro- and nanoscale texture) promote bone bonding which may be of benefit in situations where large demands are imposed on biomechanically stable interfaces, such as in early loading and in compromised conditions.

## Introduction

For more than 30 years, osseointegrated implants inserted in the temporal bone have served as an important component of bone-anchored hearing systems (BAHS) [1]. Low age and children have been diversely reported to be associated with both lower [2–4] and comparable [5] implant survival compared with an adult population. An increase in implant failures has also been reported in young patients, as well as in patients > 60 years of age, possibly related to reduced blood flow in the bone [6]. Moreover, clinical conditions such as osteogenesis imperfecta, corticosteroid medication and irradiation have been implicated in higher rates of failure [7]. For this reason, despite the overall low incidence of implant failure, ranging between 2% and 17% in mixed populations (depending on the follow-up time) [1], there is a need to understand the mechanisms of failure, as well as to explore novel measures to improve the clinical performance of these devices.

Implant surface modification is a main strategy that can optimise the surface properties of an implant and promote its integration in the recipient bone. The physical (e.g. topography) and chemical (e.g. oxide thickness) properties of an implant surface play a critical role in modulating the tissue response [8]. The addition of micron-level topography to conventionally machined surfaces greatly enlarges the surface area and improves the mechanical interlocking between the implant surface and bone. Moderately roughened surfaces permit higher stability and better clinical results [9–11]. It has been suggested that a combination of surface roughness at different distinct length scales (e.g. micron, submicron and nano) superimposed on one another benefits bone-implant bonding, particularly if these functionally graded surfaces mimic the hierarchical architecture of natural bone [12, 13]. By themselves, nanostructures on a surface could stimulate the osteogenic differentiation of mesenchymal stem cells *in vitro* [14]. Moreover, implant surfaces that incorporate well-defined nanotopography stimulate osseointegration *in vivo* [15].

The hierarchical structuring of titanium surfaces can be achieved by site-specific laser ablation [16] and the valley regions of threaded implants are believed to be associated with increased bone formation kinetics [17]. Previously, laser-modification in the thread valleys has been shown to enhance bone formation around the implant and increase the biomechanical anchorage of commercially pure (cp-Ti) [18, 19] and titanium alloy (Ti6Al4V) implants [20] compared with machined surfaces. Moreover, laser-modification in the thread valleys has been shown to promote direct contact between bone apatite and the surface oxide layer [21].

In the clinical situation, an objective measurement of implant-bone stability is required. The stiffness of the bone-implant unit can be assessed non-invasively by resonance frequency analysis (RFA), whereby oscillations are induced by a piezoelectric element and the corresponding resonance frequency is recorded. RFA values are mainly influenced by the firmness of the fixation, healing time, extent of osseointegration, stiffness of the surrounding bone and implant geometry [22]. Clinically, RFA is used with little correlative structural information relating to the integrity of the bone-implant interface. In experimental studies, it has been suggested that RFA correlates with other commonly used parameters of osseointegration, such as removal torque (RTQ) and histomorphometry of the bone-implant interface zone [23–26]. However, all these studies have used univariate correlation tests (e.g. Spearman or Pearson) that do not consider the effects of confounding factors between different parameters. Moreover, no correlative studies of the matrix composition of the interface zone with RFA and RTQ have been performed.

It has previously been demonstrated that the rabbit tibia (mainly cortical bone) or femur (mainly trabecular bone) serve as suitable experimental animal models corresponding to the clinical insertion sites in the temporal bone (mainly cortical bone) and the maxilla (mainly trabecular bone). After six to eight weeks in this model, it is possible to determine differences in bone-implant contact (BIC) [27], bone area in threads (BA) [27] and RTQ [19] between different implant surface modifications. In this work, a set of correlative techniques has been employed, including optical microscopy, electron microscopy, Raman spectroscopy, biomechanical testing and correlation and regression analyses, after eight weeks of submerged healing in the rabbit tibia. The aims were to determine (i) whether the hierarchical structuring of titanium using laser-modification promotes implant stability, (ii) whether hierarchical structuring influences the composition and ultrastructure of the surrounding bone in comparison with machined surfaces and (iii) the relationship between different parameters commonly used to characterise osseointegration (RFA at retrieval, RTQ, RFA at insertion, implant type/surface, BIC and BA).

## Materials and Methods

### 2.1 Implant design

Screw-shaped implants (diameter 3.75 mm, length 5 mm) machined from commercially pure titanium (cp-Ti, grade 4). For half the implants, selective laser ablation was employed to produce site-specific surface modification confined to the thread valley, reaching approximately 30% of the thread height on each flank, leaving the majority of the implant as machined. The laser ablation process was performed using a Q-switched Nd:YAG laser 120W (Rofin-Sinar Technologies Inc., Plymouth, USA) with 1064 nm wavelength and 125 μm spot size on rotating implants (15 RPM) in ambient air. One millimetre at each end of the implants was left untreated. The remaining half of the implants were left as machined. The implants were cleaned in Extran MA01® (Merck Millipore, Darmstadt, Germany) prior to sterile packaging and subsequent autoclaving.

### 2.2 Surface characterisation

Surface chemical analysis was performed using Auger electron spectroscopy (AES) (PHI 700 Scanning Auger Nanoprobe) on two implants of each type, where four locations were analysed on each implant. The oxide thickness was determined by AES depth profiling using two implants of each type, where three areas with dimensions of 10 μm x 10 μm were analysed on each implant. The surface morphology was evaluated by scanning electron microscopy (SEM) using a Leo Ultra 55 FEG SEM (Leo Electron Microscopy Ltd, UK) in the secondary electron mode operated at 5 kV accelerating voltage, using a regular secondary electron detector at low resolution and an in-lens detector at high resolution, at ×50–200,000 magnifications. The surface topography was analysed by white light 3D interference microscopy using a Wyko NT1100 optical profiler (Veeco Instruments Inc., Tucson, USA) using a ×20 magnification lens in the vertical scanning interferometry mode with a vertical resolution of 3 nm. Two implants of each type were used and four locations were analysed on each implant. The collected data were processed (Weeco Vision 32 v3.43) and the roughness data parameters were calculated (SPIP 3D Image Processing v3.0.0.9 software, Image Metrology A/S, Hørsholm, Denmark).

### 2.3 Animal model and surgical procedure

The experiment (including the pre-operative, operative and post-operative care and maintenance of the animals) was approved by the local Animal Ethics Committee at the University of Gothenburg, Gothenburg, Sweden (Dnr 291–2012). Ten adult female New Zealand white rabbits (Lidköpings Kaninfarm, Lidköping, Sweden; weighing 4–5 kg) received one implant of either type in each proximal tibial metaphysis for a healing period of eight weeks. The animals were kept on a 12-hour day/night cycle with ad libitum access to food and water. Prior to the surgery, the animals were anaesthetised with a 1 mL intramuscular injection of Hypnorm (fentanyl citrate 0.315 mg/mL + fluanisone 10 mg/mL, VetaPharma Ltd, Leeds UK; 0.25 mL/kg body weight) and a 1 mL intraperitoneal injection of Stesolid (5 mg/mL diazepam Actavis group hf, Hafnarfjordur, Iceland; 1.25 mg/kg body weight). Additional Hypnorm (intramuscular, 0.3 mL) was given at 20–30 minute intervals to maintain anaesthesia. Post-operatively, 0.4 mL (0.3 mg/mL) of Temgesic (Reckitt Benckiser Healthcare Ltd, Hull, UK) was administered subcutaneously. The animals received two additional 0.4 mL doses of Temgesic a day, for three days.

The surgical procedure was performed in a sterile environment. The surgical site was shaved and cleaned using chlorhexidine digluconate (5 mg/mL in 70% ethanol; Fresenius Kabi, Uppsala, Sweden). The limbs were covered with sterile drapes, leaving only the surgical area exposed. The bone surface was exposed by incision and blunt dissection of the underlying tissue, including the periosteum. The drill holes were prepared by a stepwise enlargement, starting with a 2 mm diameter round burr, followed by a 2 mm twist drill, 3 mm pilot drill and a 3 mm twist drill under copious irrigation with saline. The final preparation was made with a screw tap. The implants were installed at slow speed (15 rpm) with an OsseoSet^TM^ 200 drill unit (Nobel Biocare AG, Zurich, Switzerland). The implant stability quotient (ISQ) was measured prior to attaching the cover screw by means of resonance frequency analysis (RFA) with the Osstell Mentor system (Osstell AB, Göteborg, Sweden). Two measurements were made at 90° to each other and their mean values were considered for statistical analyses. Eight weeks post-implantation, the ISQ was measured again. To minimise measurement errors, each rabbit had its individual ISQ probe (SmartPeg^TM^, Osstell AB, Göteborg, Sweden) for measurements made at implantation and retrieval.

### 2.4 Biomechanical evaluation

The animals were euthanised by an intravenous overdose of sodium pentobarbital (60 mg/mL). A removal torque (RTQ) evaluation was made using a torque-testing machine connected to the implants via a custom fabricated adapter. The system was aligned and calibrated. The RTQ value was monitored in real time while rotating the implants at a constant angular speed of 0.2°/s.

### 2.5 Histological evaluation

The implants and surrounding bone were retrieved *en bloc* and processed by fixation in 4% paraformaldehyde, stepwise dehydration in a graded ethanol series, followed by embedding in plastic resin (LR White, London Resin Co. Ltd, UK). The embedded blocks were bisected. One half-block of each specimen was used to prepare a 50 μm thick central ground section (EXAKT® Apparatebau GmbH & Co, Norderstedt, Germany) [28] stained with toluidine blue. Qualitative histology and quantitative histomorphometry were performed, to determine the amount of bone-implant contact (BIC) and bone area (BA) within the implant threads, using light optical microscopy (Nikon Eclipse E600; Nikon NIS-Elements software).

### 2.6 Backscattered electron scanning electron microscopy

The resin embedded bone-implant blocks were wet polished with 400–4000 grit SiC grinding paper. The samples were air-dried overnight prior to low-vacuum backscattered electron scanning electron microscopy (BSE-SEM) imaging in a Quanta 200 environmental SEM (FEI Company, The Netherlands) operated at 20 kV and 0.5 Torr water vapour pressure. Five test and control pairs (from the same animal) were used. For each sample, the first thread located below the level of the original cortical bone (first endosteal thread) was imaged in order to ensure that only *de-novo*-formed bone was being analysed.

#### Mineralised bone area

The mineralised bone area (B.Ar) in the first thread located below the level of the original cortical bone was quantified on BSE-SEM images recorded at ×300 magnification. All unmineralised areas in each recorded image were excluded by segmentation using ImageJ (imagej.nih.gov/ij).

#### Osteocyte density

The osteocyte density, i.e. the average number of osteocytes per mineralised surface (N.Ot/B.Ar), was quantified on BSE-SEM images recorded at ×300 magnification. All unmineralised areas in each recorded image were excluded by segmentation using ImageJ (imagej.nih.gov/ij) and the osteocytes were quantified using the Cell Counter plug-in [29].

### 2.7 Raman spectroscopy

Raman spectroscopy was used to investigate the composition of the newly formed bone within and around the first implant thread completely filled with new bone, below the level of the original cortical bone. Spectra were collected at room temperature by a nitrogen-cooled charge-coupled device (CCD) detector connected to a Dilor XY 800 spectrometer (Horiba, Jobin Yvon GmbH), equipped with a 676 nm Ar/Kr laser operated at ~100 mW, with a 50x objective, 600 groove/mm grating, 300 mm focal length and pinhole size of 100 μm. Five test and control pairs (from the same animal), the same as for prior BSE-SEM, were used. For each sample, three spectra each were recorded in areas of mineralised bone, approximately 50–100 μm from the implant surface at the inner 1/3 (thread valley), the outer 2/3 (thread flank) and immediately outside the thread. For each sample, the first thread located below the level of the original cortical bone (first endosteal thread) was imaged in order to ensure that only *de-novo*-formed bone was being analysed.

At each position, five acquisitions were made with an integration time of 20 s each. Raw spectra were truncated between 300–1800 cm^-1^ and background fluorescence subtraction was performed by fitting a fourth-order polynomial function using the Background Correction program [30] for MATLAB R2015b (Mathworks Inc., Natick, MA). The wavenumber axis was adjusted so that the ν_1_ PO_4_^3-^ peaks in all spectra corresponded to ~959 cm^-1^. The baseline-corrected spectra were then normalised using Plot (http://plot.micw.eu/) to show equal intensities of the ν_1_ PO_4_^3-^ band in all spectra. Curve fitting, using mixed Gaussian and Lorentzian functions, and the quantification of integral areas were performed using MagicPlot (www.magicplot.com).

The investigated Raman metrics included mineral crystallinity, taken as the reciprocal of the full-width at half-maximum (1/FWHM) of the ν_1_ PO_4_^3-^ peak [31], the apatite-to-collagen ratio, also referred to as the mineral-to-matrix ratio (ν_2_ PO_4_^3-^/Amide III) [32, 33], and the carbonate-to-phosphate ratio (ν_1_ CO_3_^2-^/ν_1_ PO_4_^3-^ [34]; ν_1_ CO_3_^2-^/ν_2_ PO_4_^3-^ [35]. The integral areas were: ν_1_ PO_4_^3-^ (∼930–980 cm^-1^), ν_2_ PO_4_^3-^ (∼410–460 cm^-1^), Amide III (∼1215–1300 cm^-1^) and ν_1_ CO_3_^2-^ (∼1050–1100 cm^-1^) [36].

### 2.8 Direct visualisation of osteocyte morphology

Resin embedded bone-implant blocks were prepared for the direct visualisation of osteocytes adjacent to the implant surface using a resin cast etching procedure [37, 38]. The surface of the resin embedded blocks (the same ones previously used for BSE-SEM imaging) was wet polished with 400–4000 grit SiC grinding paper. A few selected blocks were also polished from the reverse side to expose the outer curvature of the implants and thereby the surface of the thread valley. The polished samples were sequentially immersed in 9% H_3_PO_4_ and 5% NaOCl, rinsed and allowed to air-dry overnight. The samples were Au sputter-coated (~10 nm) for high-vacuum secondary electron SEM imaging in an Ultra 55 FEG SEM (Leo Electron Microscopy Ltd, UK) operated at 5 kV accelerating voltage, 5 mm working distance and 30 μm aperture size.

### 2.9 Ultrastructural analysis

One selected block from the laser-modified group was sputter coated with Au/Pd and transferred to a dual-beam instrument, Versa 3D FIB-SEM (FEI Company, Eindhoven, The Netherlands), for the preparation of an electron transparent lamella for transmission electron microscopy (TEM) [39, 40]. The area of interest was located by secondary electron imaging and protected by ion-assisted Pt deposition. A relatively thick (3 μm) lamella was cut and lifted out by an Omniprobe, after which it was transferred to a TEM grid, for final thinning to approximately 200 nm using a gradually decreasing ion beam current. No attempt was made to prepare FIB-milled TEM specimens from the machined implants due to well-documented technical challenges [41].

Transmission electron microscopy was performed in an FEI Titan 80–300 (FEI Company, Eindhoven, The Netherlands) equipped with a Probe Cs corrector and operated at 300 kV. Bright-field TEM and high-angle annular dark field-scanning TEM (HAADF-STEM) imaging was performed to study the ultrastructure of the bone-implant interface. Further, site-specific, energy-dispersive X-ray spectroscopy (EDX) was performed across the interface zone using a nanoprobe in STEM mode.

### 2.10 Statistical analysis

The Mann-Whitney U test was used for the surface characterisation data (surface elemental composition, oxide thickness, and surface roughness). The non-parametric Wilcoxon’s signed rank test for pair-wise analysis was used for the biomechanical tests (RFA and RTQ), histomorphometry (BIC and BA) and Raman spectroscopy. Pearson correlation analysis was used to detect possible positive or negative correlations between the different test parameters of the various analyses performed.

Pearson correlation and linear regression models were used to determine the relationship between different parameters of osseointegration. Analyses were performed on merged groups, machined and laser-modified implants (pooled as *n* = 20). First, the Pearson correlation analysis was performed between parameters anticipated to measure implant stability: RTQ and RFA at retrieval (*r*RFA) and the parameters that describe the degree of bone-implant contact: BIC (inner BIC and total BIC), as well as the amount of bone filling in the implant thread: BA (inner BA and total BA). The type of inserted implant, whether machined or laser-modified, and the RFA at insertion (*i*RFA) were included. Next, parameters that demonstrated significant correlations according to the Pearson analysis were entered in a multiple, stepwise, linear regression analysis. In the multiple regression model, RTQ and *r*RFA were regarded as dependent variables, whereas the significantly correlated inner BIC, total BIC, inner BA or total BA were used as independent variables that may or may not predict the dependent variables. Whenever the implant type and/or the *i*RFA revealed a significant correlation, in the Pearson model, they were then also controlled for as confounding factors in the linear regression model.

All statistical tests were conducted using (SPSS Statistics, v.15, IBM Corporation) and *p* values *< 0*.*05* were considered statistically significant, unless otherwise indicated. Mean values ± standard deviations are presented, unless otherwise indicated.

## Results

### 3.1 Surface characterisation

Auger electron spectroscopy (AES) demonstrated a predominance of Ti, O and C on both implant surfaces, with only minor contamination by Ca and S. AES depth profiling confirmed a difference in surface oxide thickness, 53 nm for the laser-modified surface and 13.8 nm for the machined surface (Table 1).

**Table 1:** Summary of surface characterisation data (quantitative parameters) for screw-shaped implants: surface elemental composition determined by AES^†^, oxide thickness determined by AES depth profiling, and surface roughness measured by interference microscopy.

Parameter	Machined	Laser	*p*-value
C (%)	66.3 ± 2.2	62.6 ± 1.6	0.002
Ti (%)	8.4 ± 0.8	10.4 ± 0.5	0.001
O (%)	25.4 ± 1.4	26.9 ± 1.2	0.052
Oxide thickness (nm)	13.8 ± 0.7	53 ± 16.2	0.004
S_a_ (μm)	0.27 ± 0.03	3.35 ± 0.44	0.001
S_dr_ (%)	14.4 ± 2.2	88.4 ± 24.3	0.001
S_ci_	1.7 ± 0.1	1.7 ± 0.1	0.817

S_a_: arithmetic mean deviation of the surface; S_dr_: developed surface area ratio; S_ci_: surface core fluid retention index. †: The values are normalised to consider only the elements C, Ti and O.

**Fig 1:**
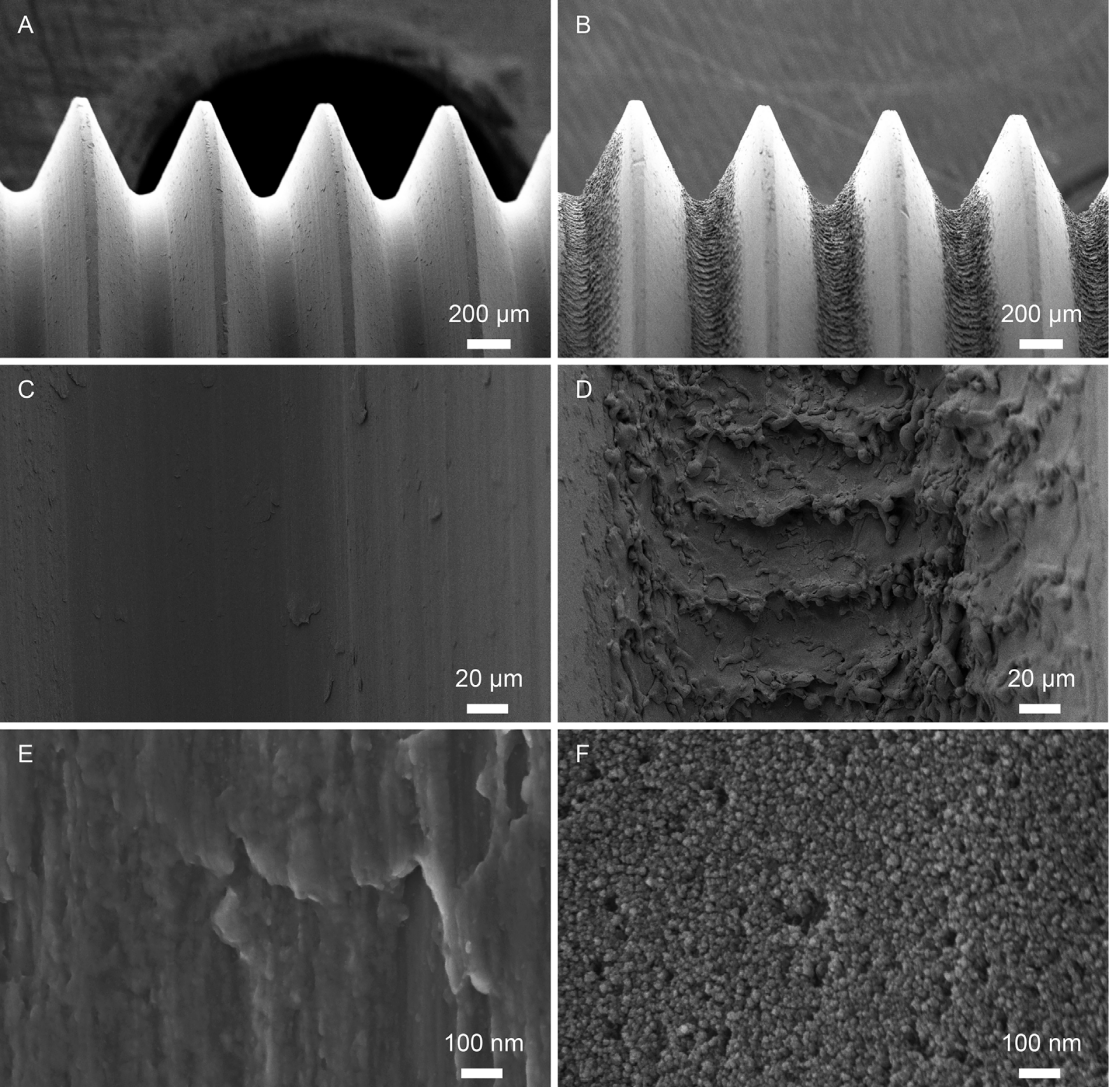
SEM images of the implant surface. Low-magnification images of the (A) machined and the (B) laser-modified implants. Surface microtopography of the (C) machined and the (D) laser-modified implants. High-magnification images show (E) ridges and grooves as remnants of the machining process for the machined implants and (F) a distinct nanotexture superimposed on the surface microtopography of the laser-modified implants.

The machined surface was relatively smooth, with a repetitive pattern of machining ridges (Fig 1A, 1C and 1E). A distinct hierarchical surface structure with a combined macro-, micro- and nanotopography was found in the thread valleys of the laser-modified surface (Fig 1B, 1D and 1F). The quantitative interferometry analysis of surface roughness on the microscale demonstrated markedly higher roughness for the laser-treated implants (Table 1).

### 3.2 Biomechanical evaluation

#### Resonance frequency analysis (RFA)

**Fig 2:**
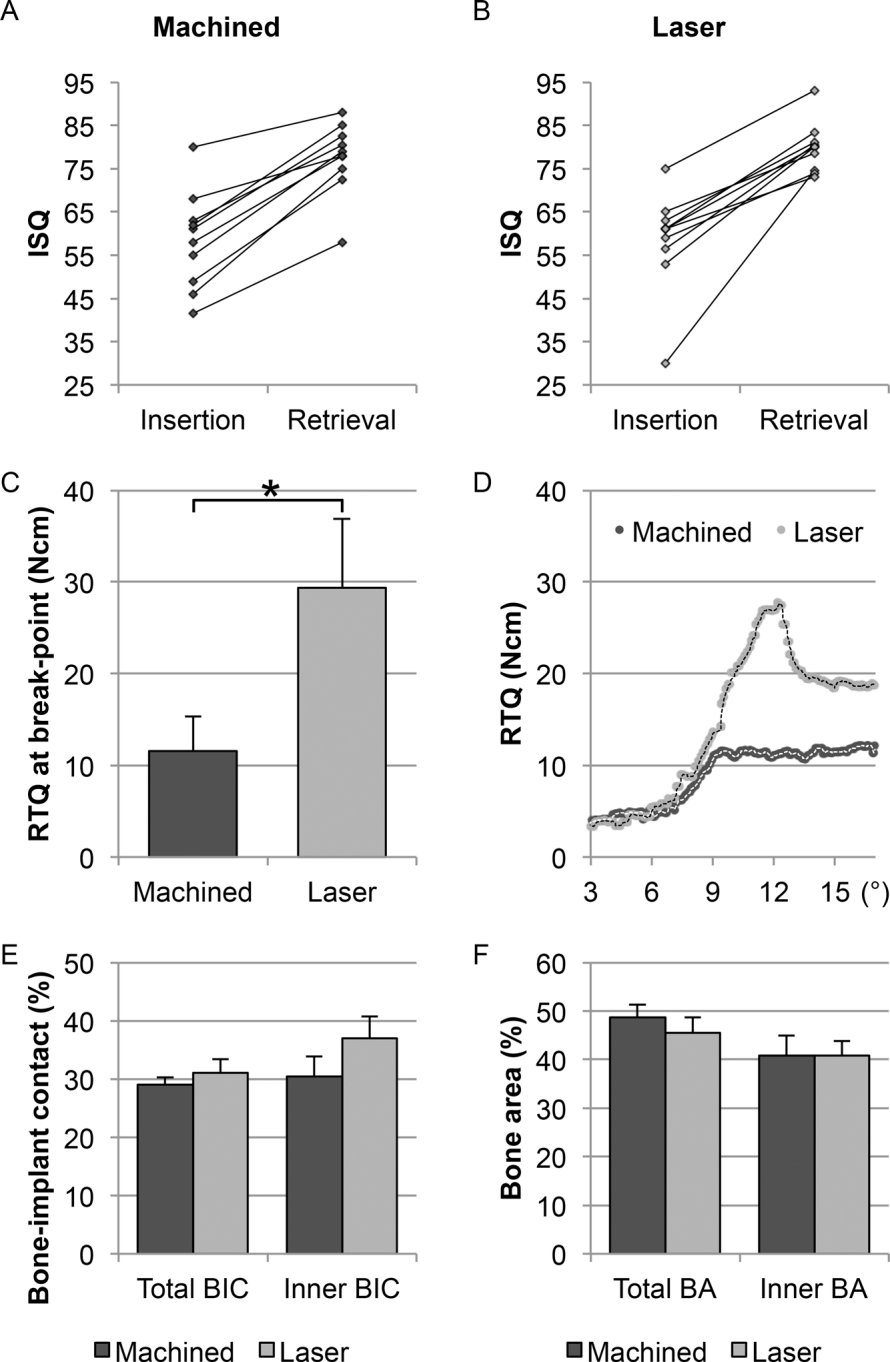
RFA, RTQ, load deformation and histomorphometry. (A-B) Resonance frequency analysis (RFA) showing changes in ISQ values over time. (C) Removal torque (RTQ) at break-point. (D) Typical load deformation curves (applied load vs. angular deformation) for the machined and the laser-modified implants showing the distinctly different patterns of mechanical failure at a constant rate of 0.2°/s. (E-F) Histomorphometry. The total bone-implant contact (BIC) and the total bone area (BA) in the threads were determined along the entire length of the threaded part of the implant. The inner BIC and the inner BA were determined in the inner 1/3 of the threads of the respective implant types.

The analysis of the implant stability quotient (ISQ) revealed significantly increased values over time between insertion (*i*RFA) and retrieval (*r*RFA) (Fig 2A and 2B). The ISQ values increased from 58 ± 11 to 78 ± 8 for the machined implants and 58 ± 12 to 80 ± 6 for the laser-modified implants (mean values ± SEM). However, neither at insertion nor at retrieval were any significant differences in ISQ values detected between the two implant types.

#### Removal torque (RTQ)

As demonstrated (Fig 2C, and S1 Fig), the laser-modified implants showed a significantly higher RTQ at break-point compared with machined implants (153% increase). Distinctly different patterns of deformation (applied torque vs. angular deformation) were observed for the two implant types (Fig 2D). The machined implants showed a moderately linear increase in torque up to a few degrees, thereafter reaching a plateau at constant or slightly increasing torque. In contrast, the load deformation plots of the removal of laser-modified implants showed a sharp torque increase, followed by a distinct break-point with a shorter or no plateau period.

### 3.3 Histological evaluation

**Fig 3:**
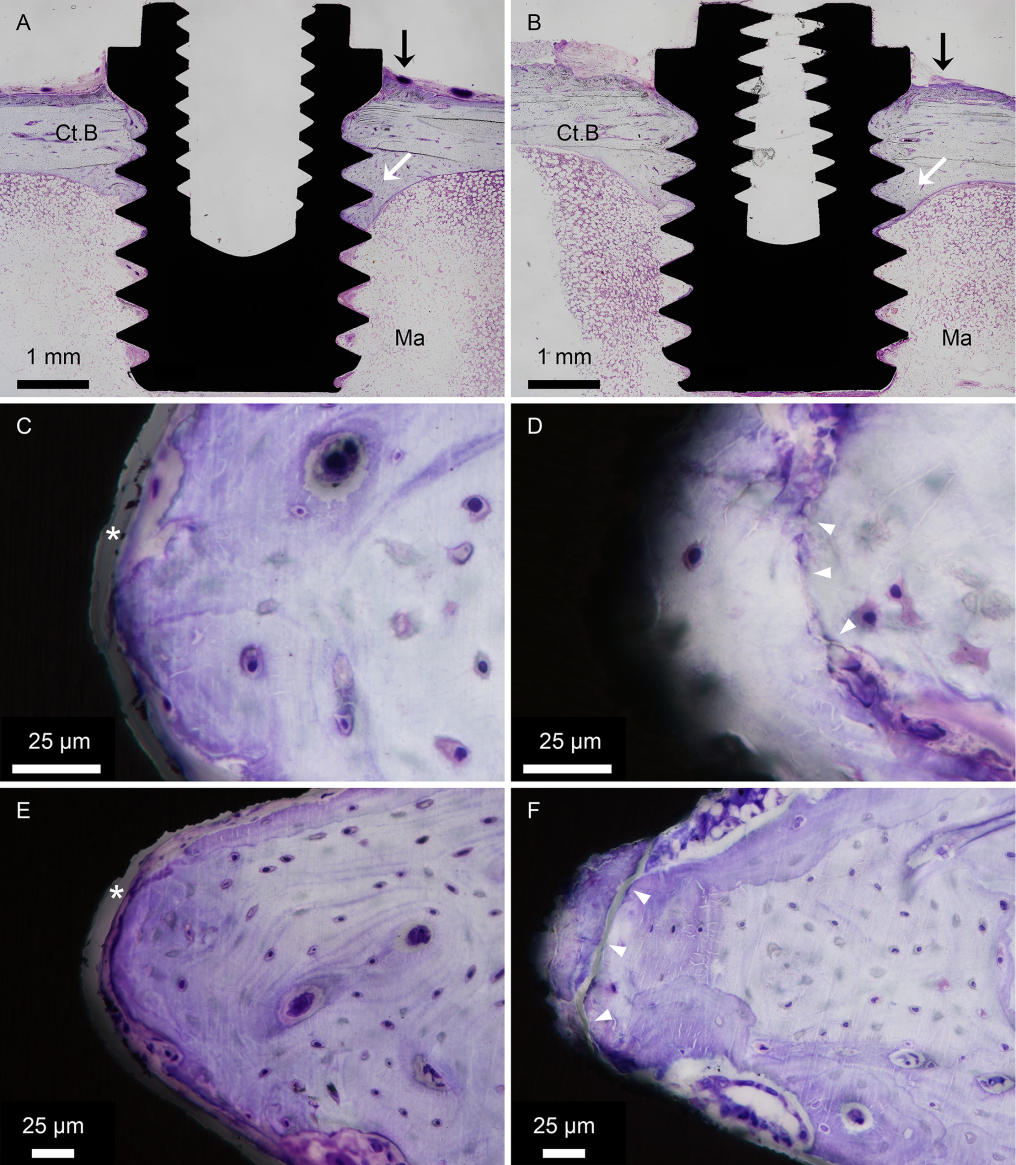
Histology. Undecalcified toluidine blue-stained sections of the (A) machined and (B) laser-modified implants. Both types of implants are vertically and unicortically positioned in the tibia. The two upper threads are located in the original cortical bone (Ct.B). Endosteal bone downgrowth (white arrows) is observed, extending downwards to the level of the third/fourth thread, whereas the remaining threads are mainly located in the bone marrow (Ma) compartment. Occasionally, periosteal bone formation (black arrows) is observed, reaching up to the implant flange yet remaining below the level of the cover screw. Bone interfacing the (C, E) machined and (D, F) laser-modified implants, at the level of the upper cortical thread (C-D) and the level of the endosteal threads (E-F). For the machined implants, a separation is frequently detected (white asterisk) between bone and implant. The laser-modified surface revealed bone in direct contact. For all laser-modified implants, fracture lines (white arrowheads) were observed in the bone within the threads at short distances (typically 30–50 μm) from the implant surface and running parallel to the implant surface in the thread valley.

Histological evaluation of the implants (subjected to removal torque) showed that they were vertically and unicortically positioned in the tibia (Fig 3A and 3B). Further, all implants, irrespective of surface modification, demonstrated an endosteal downgrowth. No adverse tissue response, including inflammation, was observed (Fig 3C–3F). At high magnification, a common characteristic of all laser-modified implants was the presence of bone fracture lines in the thread valleys, running parallel to and at a short distance (typically 30–50 μm) from the implant surface (Fig 3D and 3F). These fracture lines were detected in both the cortical and the endosteal threads. The fractured fragment of bone in the thread valley remained attached to the laser-modified part of the implant surface. These fracture lines were almost never detected in the machined implants, where a separation between the implant surface and bone was instead encountered (Fig 3C and 3E).

**Fig 4:**
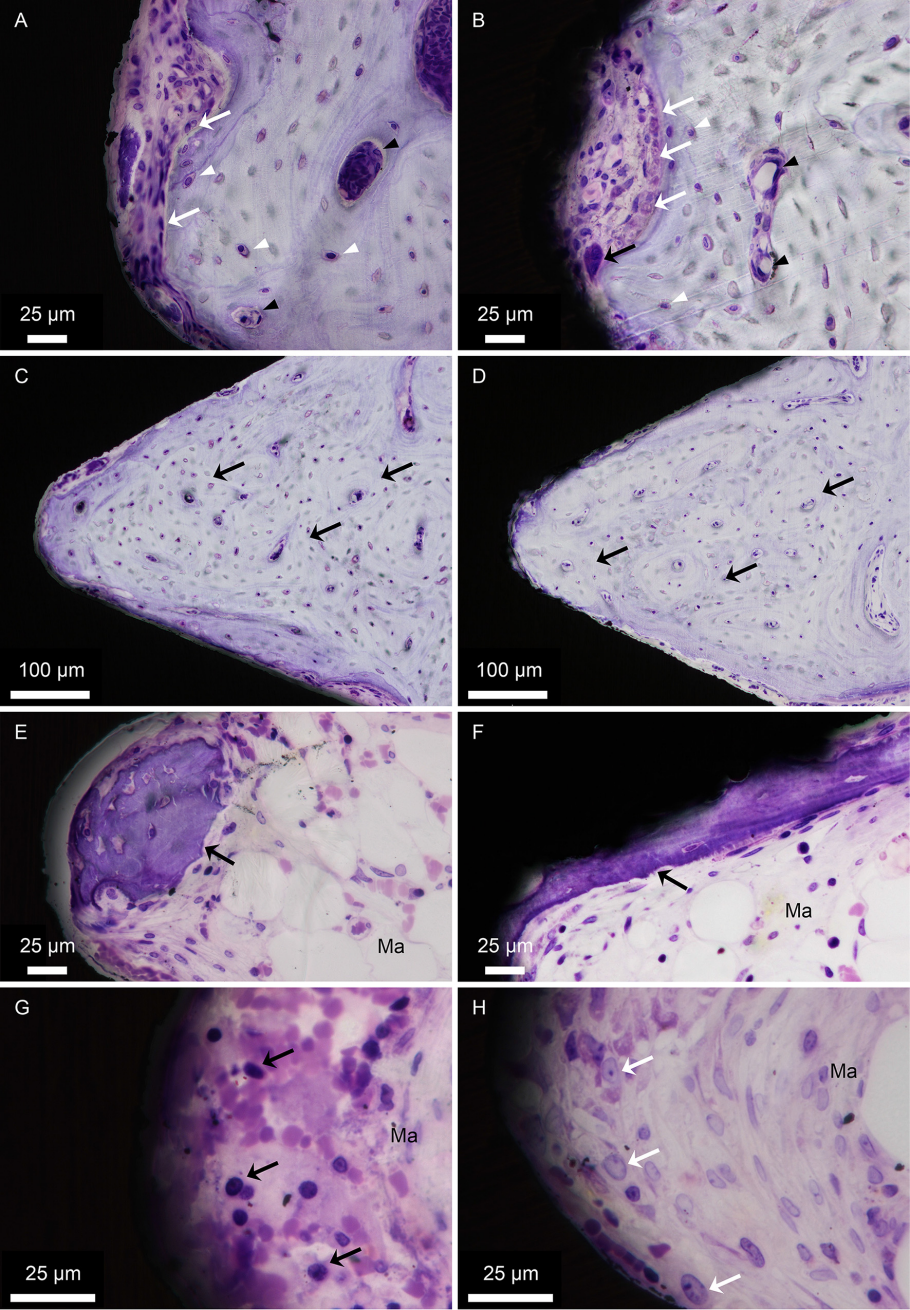
Histology. Undecalcified toluidine blue-stained sections of the (A, C, E, G) machined and (B, D, F, H) laser-modified implants. At the bone-implant interface, morphological features of bone formation and remodelling can be clearly observed in different threads in the cortical bone and the endosteal and bone marrow compartments. (A-B) Areas of ongoing remodelling. Osteoclasts (black arrows), osteoblasts (white arrows) and osteocytes (some of which are indicated by white arrowheads) are located in areas undergoing active remodelling at the interface. Blood vessels are indicated by black arrowheads. (C-D) For both implant types, mature bone occupies the endosteal threads, consisting of densely packed osteons (black arrows), with central blood vessels, surrounded by concentric bone lamellae and mature osteocytes. (E-F) Generally, the lower three threads of both implant types are occupied by bone marrow (Ma). Islands of newly formed, immature bone (black arrows) characterised by intense toluidine blue staining and large rounded osteocytes, which indicate an early stage of bone formation, are sometimes detected in the lower threads. This type of bone appears to be formed *de novo* and not as an extension from the endosteum. (G-H) Some of the bone marrow threads (Ma) show condensations of haematopoietic (black arrows) as well as relatively large and lightly stained mesenchymal-like cells (white arrows) adjacent to the implant surfaces.

For both implant types, mature bone occupied the cortical and endosteal threads, consisting of densely packed osteons, which were made up of central blood vessels surrounded by concentric bone lamellae and mature osteocytes with a typical oval shape (Fig 4A–4D). Areas of ongoing remodelling, with osteoclasts and osteoblasts, were detected for both implant types (Fig 4A and 4B). These remodelling sites were found both in the interface between bone and the implant surface and centrally in the bone filling the threads. At several locations, mature osteocytes were aligned parallel to the implant surface and sometimes at distances less than 10 μm from the implant surface.

Generally, the lower three threads of both implant types were occupied by bone marrow. However, islands of newly formed, immature bone characterised by intense toluidine blue staining and large rounded osteocytes were sometimes detected here (Fig 4E and 4F). This type of bone appeared to be formed *de novo* and not as an extension from the endosteum. Moreover, new bone was formed directly on the laser-modified surface (Fig 4F), while at a distance in the tissue thread for the machined surface (Fig 4E). In parallel with this observation, some bone marrow threads showed condensations of haematopoietic as well as relatively large and lightly stained mesenchymal-like cells (Fig 4G and 4H) for both implant types.

Quantitatively, the general histological picture was confirmed, with high levels of total bone-implant contact (Total BIC) (Fig 2E and S1 Table) and bone area (Total BA) (Fig 2F and S1 Table) for both implant types. A tendency towards higher bone-implant contact at the inner 1/3 of the implant thread (i.e. the thread valley) (Inner BIC) was noted for the laser-modified implants. No difference was observed with respect to the bone area within the inner 1/3 portion of the thread (Inner BA) between the two types of implant.

### 3.4 Correlation between biomechanical and histological analyses

In Pearson correlation analysis, the RTQ revealed a significant positive relationship with the inner BIC, total BIC and the implant type, whereas *r*RFA showed a significant positive correlation with the total BA, total BIC and *i*RFA (Table 2). When the correlated parameters were entered into the multiple stepwise linear regression model, the inner BIC was the only significant predictor of the RTQ, while the total BA was the only significant predictor of *r*RFA (Table 3). When the regression model was controlled for the confounding factors, the implant type for RTQ and the *i*RFA for the *r*RFA, it was demonstrated that the total BIC was the significant predictor of RTQ, whereas the predictive value of the total BA for the *r*RFA was eliminated (Table 4).

**Table 2:** Pearson correlation analysis between different parameters of osseointegration. The data are pooled for the machined and laser-modified implants (*n* = 20). The table shows the correlation coefficients, *r*, and the statistical significance level, *p*.

Parameter		*i*RFA	Implant type	Total BIC	Inner BIC	Total BA	Inner BA
RTQ	*r*	0.147	0.846**	0.522*	0.548*	0.201	0.228
	*p*	0.536	0.000003	0.018	0.012	0.395	0.334
*r*RFA	*r*	0.704**	0.157	0.580*	0.293	0.676**	0.326
	*p*	0.001	0.508	0.007	0.210	0.001	0.161

* = significance at *p < 0*.*05*

** = significance at *p < 0*.*005*

RTQ = removal torque measurement; *r*RFA = resonance frequency analysis at retrieval; *i*RFA = resonance frequency analysis at insertion; BIC = bone-implant contact; BA = bone area

**Table 3:** Multiple stepwise linear regression model without controlling for the confounding factors. The data are pooled for the machined and laser-modified implants (*n* = 20).

Dependent variables	Independent variables	Adjusted R^2^	β-coefficient	*p*-value
RTQ	Inner BIC	0.26	0.55	0.012
	Total BIC	0.26	0.26	0.375
*r*RFA	Total BA	0.43	0.68	0.001
	Total BIC	0.43	0.18	0.499

RTQ = removal torque measurement; *r*RFA = resonance frequency analysis at retrieval; BIC = bone-implant contact; BA = bone area

**Table 4:** Multiple stepwise linear regression model controlled for the confounding factors. The data are pooled for the machined and laser-modified implants (*n* = 20).

Dependent variables	Independent variables	Controlled confounders	Adjusted R^2^	β-coefficient	*p*-value
RTQ	Inner BIC	Implant type	0.84	0.11	0.46
	Total BIC	Implant type	0.84	0.38	0.001
*r*RFA	Total BA	*i*RFA	0.47	0.38	0.078
	Total BIC	*i*RFA	0.47	0.292	0.141

RTQ = removal torque measurement; *r*RFA = resonance frequency analysis at retrieval; *i*RFA = resonance frequency analysis at insertion; BIC = bone-implant contact; BA = bone area

### 3.5 Backscattered electron scanning electron microscopy

**Fig 5:**
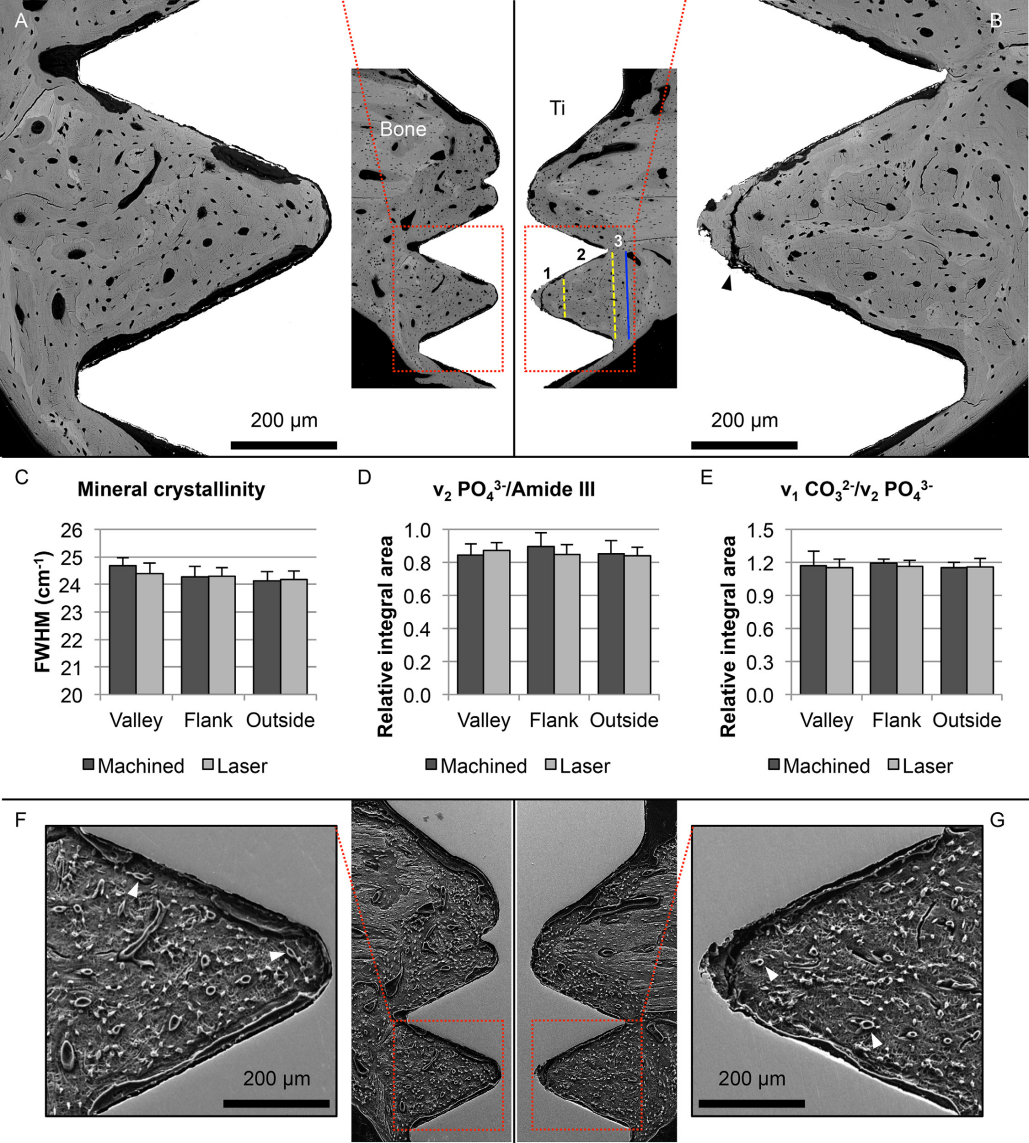
Backscattered electron scanning electron microscopy (BSE-SEM), Raman spectroscopy and resin cast etching. (A-B) BSE-SEM images of the (A) machined and the (B) laser-modified implants, showing mature, remodelled, osteonal, lamellar bone filling the implant threads. Following biomechanical testing (RTQ), bone detaches from the machined implant surface, resulting in a separation at the bone-implant interface, while a fracture line appears in the bone at some distance from the laser-modified implant surface (black arrowhead). (C-E) Raman metrics evaluated at the level of the first endosteal thread (as indicated in the insert image in B); 1: valley (corresponding to the inner 1/3 of the thread), 2: flank (corresponding to the outer 2/3 of the thread), 3: outside. (F-G) The same threads (as in A-B) are visualised following resin cast etching to confirm the presence of vasculature in the vicinity of the implant surface. The osteocyte lacuno-canalicular network (Ot-LCN) communicates with Haversian canals (white arrowheads), as well as extending towards the implant surface.

The newly formed bone within the implant threads was highly mineralised (Fig 5A and 5B and S2 Fig), but it exhibited relatively lower BSE *Z* (atomic number) contrast than the original cortical bone. The endosteal threads containing only *de-novo*-formed bone showed as much as 80 ± 3% and 82 ± 4% mineralised bone area for the machined and laser-modified surfaces, respectively. Furthermore, the osteocyte densities within these threads were also comparable, 1019 ± 118 mm^-2^ and 1005 ± 126 mm^-2^ for the machined and laser-modified surfaces, respectively.

### 3.6 Bone composition

At each analysed location, i.e. the valley, flank and outside (Fig 5C–5E and S3, S4 and S5 Figs), the mineral crystallinity (1/FWHM ν_1_ PO_4_^3-^), the apatite-to-collagen (ν_2_ PO_4_^3-^/Amide III), and the carbonate-to-phosphate (ν_1_ CO_3_^2-^/ν_1_ PO_4_^3-^, and ν_1_ CO_3_^2-^/ν_2_ PO_4_^3-^) ratios were comparable for the two implant types.

### 3.7 Direct visualisation of osteocyte morphology

**Fig 6:**
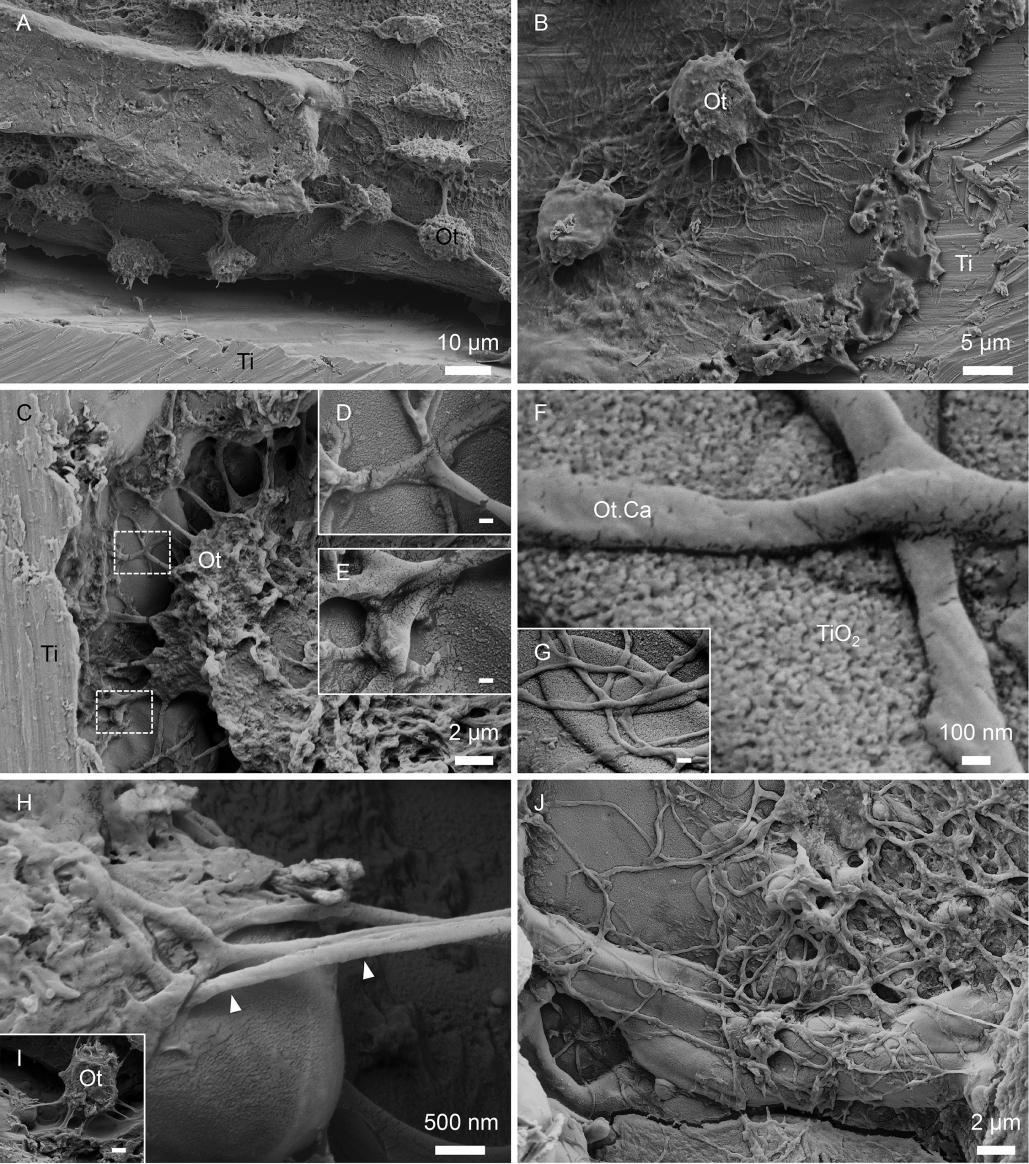
Osteocyte communication with the implant surface. Direct visualisation of osteocyte (Ot) morphology adjacent to the (A-B) machined and the (C-J) laser-modified implant surfaces following resin cast etching. (A-B) Osteocytes are observed adjacent to the machined implant surface (Ti). (C-E) An osteocyte is seen in close proximity to the laser-modified surface (Ti). (F-G) Osteocyte canaliculi are attached to the surface TiO_2_ layer. (H-I) Osteocyte canaliculi (white arrowheads) extend approximately 6 μm to approach a globular feature within the laser-ablated part of the implant surface. (J) Osteocyte canaliculi form a vast intercommunicating network directly on the laser-modified implant surface. Scale bars in D and E = 200 nm, G = 500 nm and I = 2 μm.

Following resin cast etching, the vast osteocyte lacuno-canalicular network (Ot.LCN) could be visualised (Fig 5F and 5G). Osteocytes were found in close proximity to the implant surface and aligned parallel to it. Canaliculi were observed approaching the implant surface as well as Haversian canals. Osteocytes were aligned adjacent to the machined implant surface (Fig 6A and 6B), but the mechanical disruption of the interface due to the removal torque analysis precluded any observation of osteocyte canaliculi (Ot.Ca) directly attached to the implant surface. On the other hand, canaliculi were found in high numbers adjacent to the laser-modified implant surface (Fig 6C–6J). These canaliculi appeared to branch (Fig 6D and 6E) in close proximity to the nanotextured surface oxide layer (Fig 6F and 6G), and extended several micrometres to approach globular features on the implant surface (Fig 6H and 6I), forming an extensive intercommunicating network closely adhering to the complex microtopography of the laser-ablated areas (Fig 6J).

### 3.8 Ultrastructural analysis

**Fig 7:**
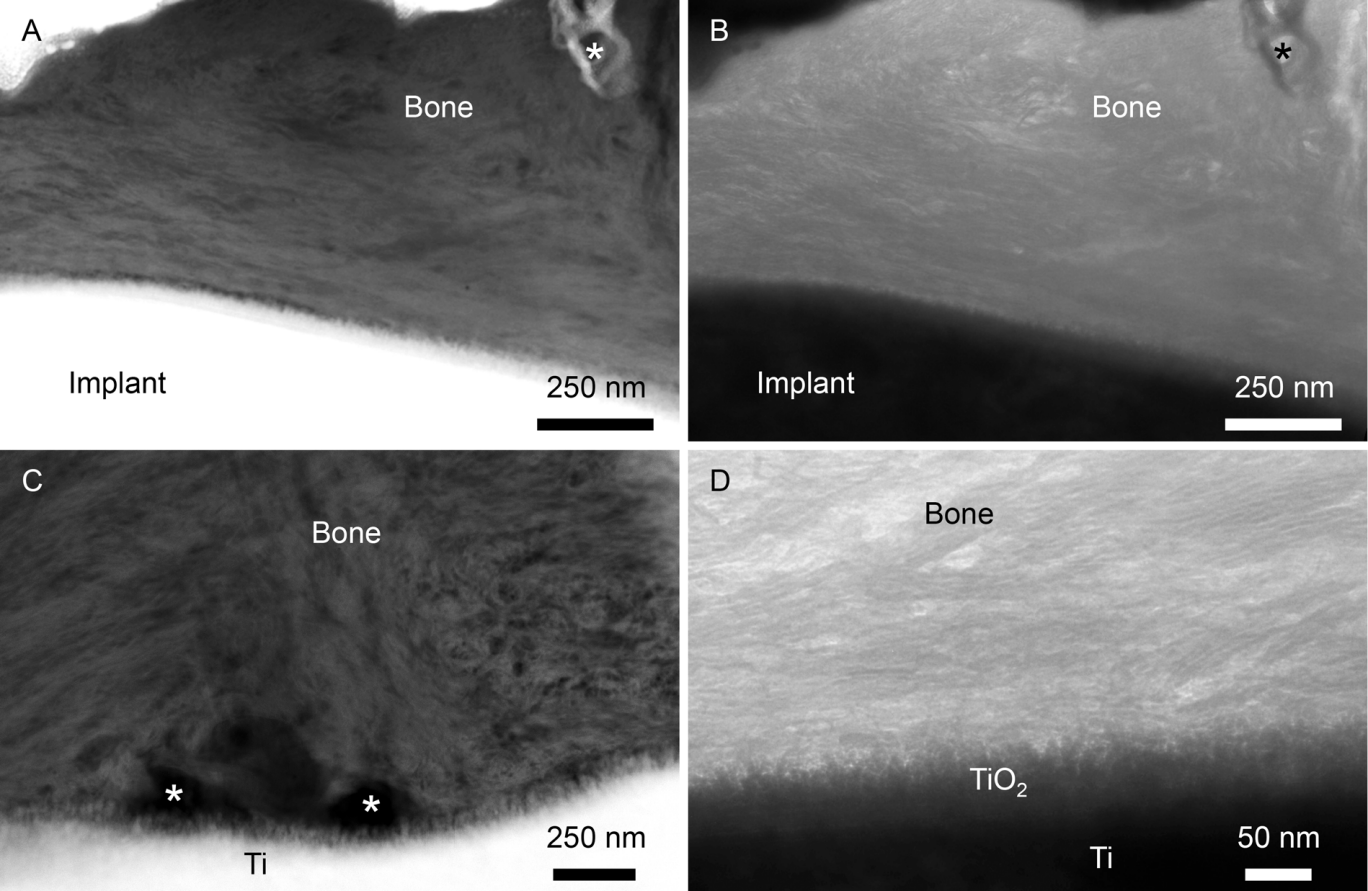
Ultrastructure. (A, C) HAADF-STEM and (B, D) TEM images demonstrating nanoscale interlocking of bone with the laser-modified implant surface. (A-B) Collagen fibrils are aligned parallel to the implant surface and osteocyte canaliculi are observed in close proximity to the implant surface (asterisks). (C) Osteocyte canaliculi appear to make direct contact with the implant surface (asterisks). (D) At high resolution, mineralised collagen fibrils interlock with the nanotextured surface TiO_2_ layer.

HAADF-STEM imaging revealed a 50 nm thick surface oxide layer with a distinct nanostructure in the cross-sectional view. Highly mineralised bone tissue was found in direct contact with this surface oxide layer (Fig 7A and 7C). Collagen fibrils were aligned parallel to the implant surface, with a characteristic 67 nm cross-banding pattern. Round (approximately 200 nm diameter) structures of low atomic number contrast, believed to be canaliculi, were present at the immediate interface and at some distance from the surface oxide. Adjacent to these structures, the collagen organisation was less regular, with a sudden shift in orientation/directionality (Fig 7C), from being parallel to the plane of view to being perpendicular to the plane of view. At high resolution, mineralised collagen fibrils appeared to interlock with the surface TiO_2_ layer (Fig 7D).

## Discussion

In the present study, one main observation was a significantly higher biomechanical anchorage, as determined by RTQ measurements, of laser-modified titanium screws in comparison with machined implants. In the search for an explanation of this observation, we evaluated the amount of new bone and the bone-implant contact (using light microscopy), the extracellular matrix composition (using Raman spectroscopy) and the structure at the implant surface (using SEM and TEM), as well as the stiffness of the bone-implant unit (by RFA).

RTQ measurements revealed a completely different load-deformation curve for laser-modified implants compared with the machined implants. The attainment of higher applied torque at break-point and the abrupt loss of strength indicate a fracture-type failure pattern. A structural correlate to this failure pattern was suggested from histological and BSE-SEM observations, showing the appearance of fracture lines in the bone, approximately 30–50 μm from the laser-modified surface. Taken together, these findings indicate that the interfacial adhesion between the implant surface and bone was stronger than the bone itself. In agreement with previous observations [19, 20], the fractured fragments of mineralised tissue remained firmly attached to the surface of the laser-modified implant. In contrast, a separation was observed at the bone-implant interface for the machined implants, indicating minimal mechanical interlocking between the bone and the implant surface.

An alternative explanation of the marked difference in biomechanical anchorage could be a difference in the quality of bone formed at the implant surfaces. Histology and BSE-SEM imaging showed that the newly formed bone, particularly within the endosteal threads (of both implant types) and therefore formed *de novo*, comprised remodelled, osteonal, lamellar bone. These endosteal threads contained a high proportion of well-mineralised tissue, irrespective of the type of implant. Further, the molecular structure of bone, evaluated by Raman spectroscopy, had a similar appearance for both implant surfaces. The degree of tissue maturation was comparable for the machined and the laser-modified implant surfaces, with respect to mineral crystallinity, the apatite-to-collagen ratio and the carbonate-to-phosphate ratio. Collectively, the quality of the bone formed within the threads, as related to the composition and structural organisation of the tissue, was similar for the two types of implant and most probably did not constitute a determinant of the observed difference in RTQ. It must be underlined that it was not possible to perform a thorough characterisation of the interfacial tissue directly adjacent to the implant surface (i.e. the first 5–10 μm) due to the prior biomechanical testing and, for this reason, local variations in extracellular matrix composition as a function of implant surface properties, as reported in other studies [29, 37], could not be analysed.

A structural cue for the strong biomechanical anchorage of the laser-modified implants was instead provided by high-resolution analysis (using TEM and HAADF-STEM imaging modes) of the implant-bone interface. The bone-implant interface showed well-mineralised collagen fibrils aligned parallel to the implant surface, closely following the micro-scale surface contour. This ultrastructural observation corroborates previous observations in humans [38, 42] and animals [19, 20]. Round (approximately 200 nm diameter) structures of low atomic number contrast, presumably osteocyte canaliculi [43], were present both at a distance and in a direct contact with the oxide layer of the laser-modified surface. These structures observed using TEM closely matched the lateral dimensions of osteocyte canaliculi exposed via resin cast etching. Visualisation of the Ot.LCN following resin cast etching confirmed that the osteocytes were aligned parallel to the implant surface, with canaliculi approaching the implant surface. Attributable to biomechanical testing, the mineralised tissue had separated from the implant surface and the embedding resin subsequently infiltrated the resulting gap. In most cases, the osteocytes closest to the implant surface therefore appear to contact a very thin intervening layer of embedding resin, rather than making direct contact with the implant surface, as demonstrated previously using this technique [37]. However, the presence of osteocytes in such close contact with the implant surface suggests the ability of osteocytes to function as mechanical load-sensing components in bone.

For both implant types, the ultrastructural findings indicate that the tissue side of the bone-implant interface is in fact mineralised bone. Furthermore, the close adaptation of the well-organised, mineralised bone to the laser-modified surface suggests direct bone bonding to the implant surface and provides a possible explanation for the increase in RTQ and its associated load-deformation pattern. This functionally graded interface could therefore be regarded as the next level of osseointegration, rather than a passive, mechanically interlocked cement-line matrix. It was not, however, possible directly to compare the organisation of the bone-implant interface with the machined surface. It is well documented that sample processing procedures, e.g. fixation, dehydration and resin embedding, induce tissue distortion [43]. At the bone-implant interface, particularly for smooth, machined surfaces, the tissue shrinks during sample processing, resulting in a separation artefact a few micrometres wide. This precluded the use of FIB-SEM for preparing electron transparent specimens of the intact, machined implant-bone interface for TEM, thereby excluding the opportunity to provide an ultrastructural correlate to the relatively lower RTQ [41, 44].

Since the present study aimed to elucidate the biomechanical and morphological correlates of osseointegration, i.e. the relationships between commonly used techniques to evaluate osseointegration, no emphasis was placed on the cellular and molecular mechanisms and this could be regarded as a limitation of the work. In order to acquire a further understanding of the underpinning mechanisms, additional tools such as quantitative polymerase chain reaction (qPCR), immunohistochemistry, protein analysis and so on need to be applied. Moreover, studies of this kind will enable an understanding of the temporal course of the cellular response to machined and laser-modified surfaces.

Although not proven, one possible explanation of the increase in RTQ and the ultrastructural bonding of laser-modified implants is the increased thickness of the surface oxide layer. The oxide film is critical for implantable biomaterial applications of titanium, as it limits the release of ionic or molecular Ti species from the surface, thereby protecting the biological environment from the extremely reactive Ti metal [45]. While the composition and distribution of the oxides impart corrosion resistance in the aggressive physiological environment, the oxidation of the surface also promotes wear resistance [46].

Early studies of the role of surface oxide indicated that implant surface modification with respect to increased oxide thickness results in a high degree of bone contact [47]. Moreover, studies showed that the anodic oxidation of electropolished titanium surfaces, which produced thicker surface oxide and areas of increased roughness on a sub-micron scale, had an enhancing effect on the rate of bone formation [17]. Subsequent studies of implants with the combination of a thick oxide layer (up to 7.5 μm) and micron- and sub-micron scale roughness and porosities revealed that these implants promote the cellular and molecular events for bone formation and remodelling which result in higher BIC and higher RTQ compared with machined implants [48–50].

This work demonstrates the osseointegration of commercially pure titanium implants with site-specific laser ablation within the deepest part of the implant threads to achieve improved biomechanical anchorage. Laser ablation in the inner 1/3 of the implant thread enabled the site-specific restructuring of the implant surface, inducing the development of a distinct microtopography comprising 1–10 μm diameter globules of resolidified metal, which predictably enlarge the surface area several times over. Superimposed on this microtopography is a relatively thick (compared with machined implants) nanostructured titanium dioxide layer, which further enhances the osteoconductive/bioactive potential of the laser-modified implant surface. The macro-shape of the screw, a distinct microtopography and the fine nanotexture give rise to a three-level surface topography, thereby matching the building blocks of bone in its hierarchical architecture.

The clinical context in which the present observations will be implemented is the improvement of the bone fixation of bone-anchored hearing aids. Although implant loosening predominantly occurs during the first year [4], recent studies have reported that the survival rate of newer, wide-diameter, osseointegrated, bone-anchored hearing implants up to three years is > 96% in adult patients [51–53]. However, the incidence of implant failure due to trauma or the loss of osseointegration in paediatric patients is still fairly high, despite a two-stage surgical procedure in which the implant is only loaded after an initial submerged healing period [4, 5, 54]. The recent development of minimally invasive surgical techniques, whereby the tissue surrounding the abutment is left intact, requires longer abutments [55, 56]. In addition, candidates previously regarded as unsuitable for reasons such as irradiation [57, 58], osteogenesis imperfecta [59], diabetes mellitus [60] and Down syndrome [61] are currently being successfully treated with BAHS. Moreover, patients are given access to sound soon after surgery, with loading times as early as two weeks [62, 63]. All these factors necessitate greater implant stability in both the short and the long term.

Based on the parameters that are commonly used to support the functional and clinical assessment of implant stability (i.e. osseointegration), we hypothesised that the RTQ and the RFA at retrieval (*r*RFA) correlated to implant type, RFA at insertion (*i*RFA), total BIC, inner BIC, total BA and inner BA. Interestingly, the RTQ showed a significant correlation with the implant type, which supports the comparative analysis showing a higher RTQ for the laser-modified surface vs. the machined surface. Moreover, an association was also revealed between the RTQ and the bone-implant contact (total and inner BIC). According to the linear regression model, when excluding the effect of surface modification (i.e. the implant type), the total BIC explained about 84% of the increase in RTQ. This means that, when evaluating osseointegration, irrespective of the type of implant surface modification, the degree of total bone-implant contact, as measured by histomorphometry, is a major predictor of RTQ and thereby of implant stability. Finally, when the effect of surface modification was not excluded, the linear regression model mainly showed that the inner BIC was a predictor of RTQ.

Another important observation was that the *r*RFA correlated with the *i*RFA, total BA and total BIC but not the implant type, which is also in agreement with the comparative analysis. In the linear regression model, when the effect of the *i*RFA was not excluded, the analysis showed that *r*RFA is mainly dependent on the amount of bone contained within the threads (i.e. total BA).

In overall terms, the results suggest that the RTQ is a method that is able to discriminate between the degree of osseointegration as influenced by differences in implant surface properties. On the other hand, RFA at retrieval only reflects the amount of mineralised bone within the implant threads (BA) but not the actual adaptation of the bone to the implant surface contour (i.e. BIC).

## Conclusions

The present study shows that osseointegration was attained for two macroscopically similar yet differently structured titanium surfaces. Bone-implant contact, bone area in threads, extracellular matrix composition and osteocyte densities were similar for the machined and laser-modified implants. In contrast, laser-modified surfaces demonstrated a higher biomechanical torque, a distinctly different load-deformation pattern and a fractured interface in the surrounding bone rather that at the implant-tissue interface during torque. Taken together with the ultrastructural observation of a highly ordered arrangement of collagen fibrils aligned parallel to the surface, these findings suggest that the physico-chemical surface properties of laser-modified surfaces (thicker oxide, micro- and nanoscale texture) promote bone bonding. Attaining ultrastructural and biomechanical effects of this kind is likely to be of benefit in situations where large demands are imposed on biomechanically stable interfaces, such as in early loading and compromised conditions. From a methodological perspective, the present results also suggest that removal torque analysis has greater predictability, when it comes to the degree of osseointegration and implant stability, than resonance frequency analysis.

## Supporting Information

S1 Fig Removal torque (RTQ) values for each animal. (PDF)

S2 Fig Overview BSE-SEM images of the (a-e) machined and (f-j) laser-modified implants.
(PDF)

S3 Fig Raman spectra recorded at the valley regions of the (top) machined and (bottom) laser-modified implant threads.
(PDF)

S4 Fig Raman spectra recorded at the flank regions of the (top) machined and (bottom) laser-modified implant threads.
(PDF)

S5 Fig Raman spectra recorded outside the (top) machined and (bottom) laser-modified implant threads.
(PDF)

S1 Table Quantitative histomorphometry. (PDF)
