# Supplementary figures and images for: Laser-Modified Surface Enhances Osseointegration and Biomechanical Anchorage of Commercially Pure Titanium Implants for Bone-Anchored Hearing Systems
Source: PLoS One. 2016 Jun 14;11(6):e0157504. doi: 10.1371/journal.pone.0157504 (PMC4907497; doi:10.1371/journal.pone.0157504)

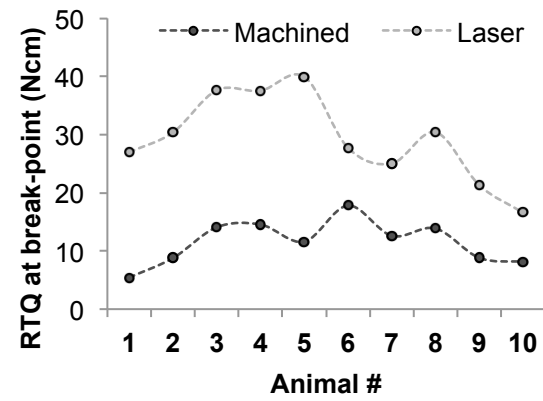

**S1 Fig. Removal torque (RTQ) values for each animal.**

Supplement: S1 Fig — (PDF) [file pone.0157504.s001.pdf]

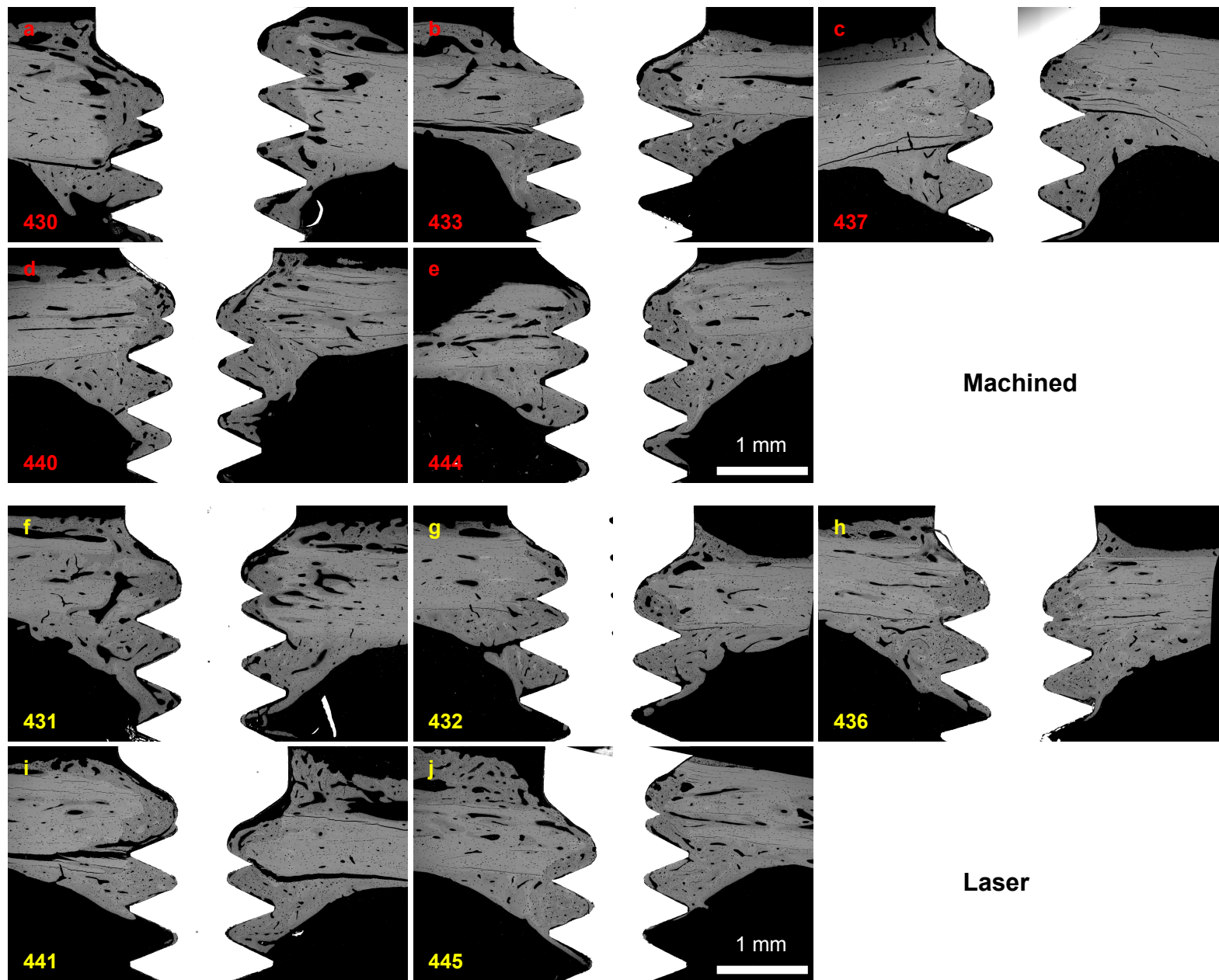

**S2 Fig. Overview BSE-SEM images of the (a-e) machined and (f-j) laser-modified implants.**

Supplement: S2 Fig — Overview BSE-SEM images of the (a-e) machined and (f-j) laser-modified implants. (PDF) [file pone.0157504.s002.pdf]
