# Supplementary material for: Laser-Modified Surface Enhances Osseointegration and Biomechanical Anchorage of Commercially Pure Titanium Implants for Bone-Anchored Hearing Systems
Source: PLoS One. 2016 Jun 14;11(6):e0157504. doi: 10.1371/journal.pone.0157504 (PMC4907497; doi:10.1371/journal.pone.0157504)
Supplement: S3 Fig — Raman spectra recorded at the valley regions of the (top) machined and (bottom) laser-modified implant threads. (PDF) [file pone.0157504.s003.pdf]

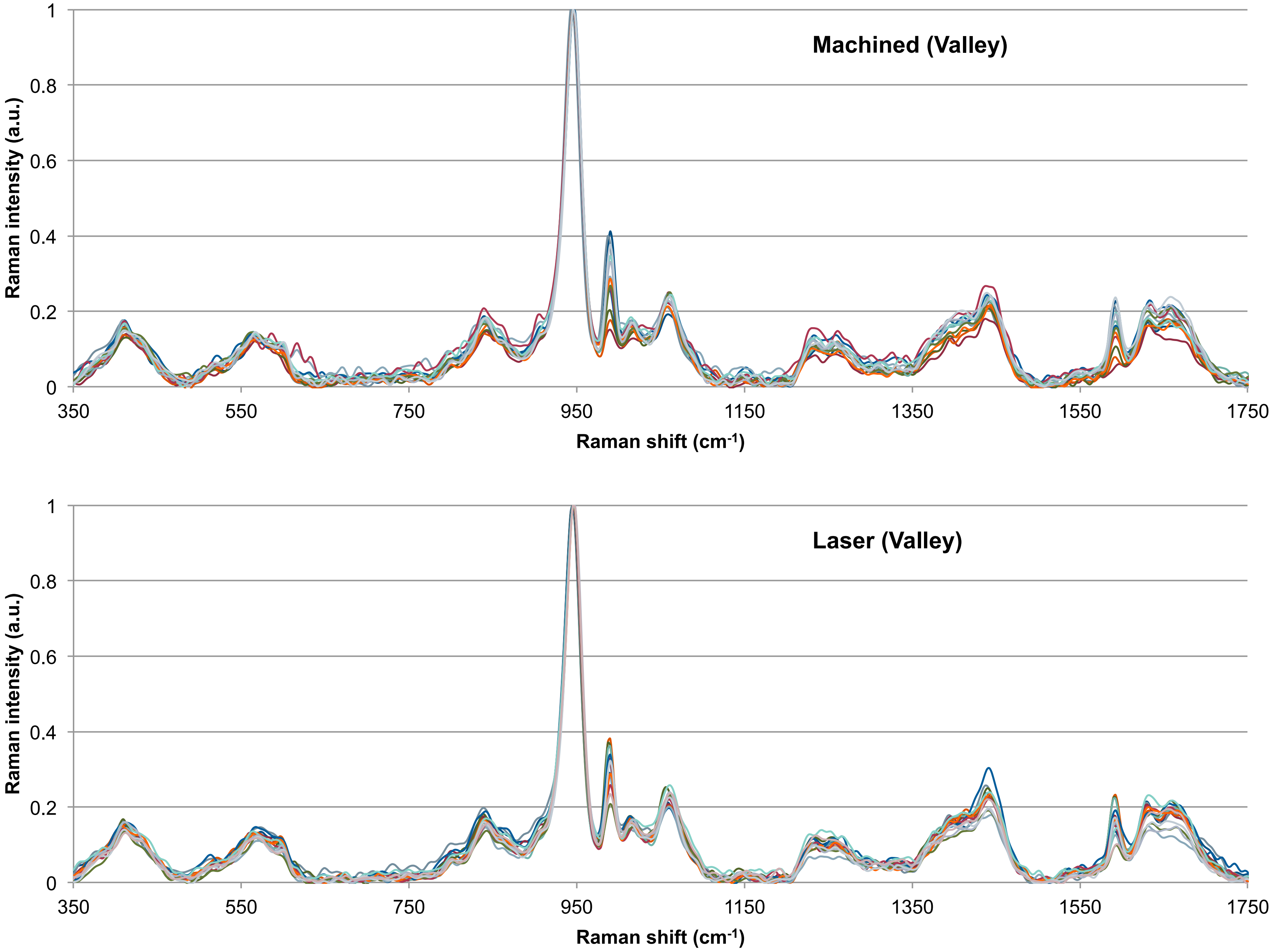

S3 Fig. Raman spectra recorded at the valley regions of the (top) machined and (bottom) laser-modified implant threads.
