# Supplementary material for: Laser-Modified Surface Enhances Osseointegration and Biomechanical Anchorage of Commercially Pure Titanium Implants for Bone-Anchored Hearing Systems
Source: PLoS One. 2016 Jun 14;11(6):e0157504. doi: 10.1371/journal.pone.0157504 (PMC4907497; doi:10.1371/journal.pone.0157504)
Supplement: S1 Table — (PDF) [file pone.0157504.s006.pdf]

	Machined					Laser				
**Rabbit #**	**Implant ID**	**Total BA (%)**	**Inner BA (%)**	**Total BIC (%)**	**Inner BIC (%)**	**Implant ID**	**Total BA (%)**	**Inner BA (%)**	**Total BIC (%)**	**Inner BIC (%)**
1	430	46	27.9	24.6	18.6	431	49.1	53.6	41	55.3
2	433	43.4	26.5	28.4	14.7	432	52.6	41.8	35.8	43.1
3	434	51.6	43.5	29.3	41	435	51.3	46	37.8	43.5
4	437	46.3	32.6	28.1	27.8	436	46.6	36.8	34.5	41.7
5	439	47.7	46.3	34.2	43.1	438	64.6	52.7	39.6	49.2
6	440	51.7	40.2	34.6	30.4	441	37.7	40	23.7	33.4
7	443	47.2	38.1	28.6	36.2	442	42.9	23.5	28.8	17.1
8	444	69.5	71.8	33.8	45.1	445	43.8	46.1	25.8	33.8
9	447	39.3	44.6	22.4	31.3	446	29.5	38	20.8	27.9
10	448	44.6	37.8	26	15.6	449	37.5	29.1	23.3	24.5
**Mean (%)**		48.7	40.9	29	30.4		45.5	40.8	31.1	37
**± SEM (%)**		2.6	4	1.3	3.6		3.1	3	2.4	3.7

BA = Bone area; BIC = Bone-implant contact

S1 Table. Quantitative histomorphometry.
